# Draft Genome Sequence of Xylella fastidiosa subsp. *fastidiosa* Strain IVIA5235, Isolated from Prunus avium in Mallorca Island, Spain

**DOI:** 10.1128/MRA.01222-18

**Published:** 2018-10-11

**Authors:** Blanca B. Landa, María P. Velasco-Amo, Ester Marco-Noales, Diego Olmo, María M. López, Inmaculada Navarro, Adela Monterde, Silvia Barbé, Miguel Montes-Borrego, Miguel Román-Écija, Maria Saponari, Annalisa Giampetruzzi

**Affiliations:** a Consejo Superior de Investigaciones Científicas, Institute for Sustainable Agriculture, Córdoba, Spain; b Centro de Protección Vegetal y Biotecnología, Instituto Valenciano de Investigaciones Agrarias, Moncada, Spain; c Serveis de Millora Agrària, Govern Balear, Palma de Mallorca, Spain; d Consiglio Nazionale delle Ricerche, Istituto per la Protezione Sostenibile delle Piante, Bari, Italy; e Dipartimento di Scienze del Suolo della Pianta e degli Alimenti, Università degli Studi di Bari Aldo Moro, Bari, Italy; Queens College

## Abstract

We report the complete annotated genome sequence of the plant-pathogenic bacterium Xylella fastidiosa subsp. *fastidiosa* strain IVIA5235. This strain was recovered from a cherry tree in Mallorca, Spain.

## Abstract (precis)

We report the complete annotated genome sequence of the plant-pathogenic bacterium Xylella fastidiosa subsp. *fastidiosa* strain IVIA5235.

## ANNOUNCEMENT

The quarantine bacterium Xylella fastidiosa recently emerged as a serious threat to the European and Mediterranean agriculture and landscape, with several outbreaks detected in Italy, France, and Spain (1). The first finding of the bacterium in Spain occurred in 2016, when cherry plants (Prunus avium) and Polygala myrtifolia were reported to be infected by strains of X. fastidiosa subsp. *fastidiosa* and *multiplex* (2). Since then, numerous outbreaks of the bacterium have been reported in the major Balearic Islands (Mallorca, Menorca, and Ibiza), where more than 15 host species have been found to be infected by strains of different subspecies of X. fastidiosa (1).

Although infections on cherry have been reported since 2014 in Italy, associated with a strain of X. fastidiosa subsp. *pauca* (3), the infections detected in Mallorca in 2016 raised major concerns, being associated with strains of X. fastidiosa subsp. *fastidiosa*. In fact, initial genetic characterization of X. fastidiosa infecting cherry in Mallorca revealed the presence of a strain harboring sequence type 1 (ST1) (2), a genotype commonly detected in strains of X. fastidiosa subsp. *fastidiosa* causing Pierce’s disease in North America. More recently on the same island, isolates harboring ST1 have been reported in several additional hosts, including grapes (1). As such, these findings triggered a series of alarms for the potential risk that the spread of this bacterium could represent for the whole European Union grape industry. In this report, we describe the draft genome sequence of X. fastidiosa subsp. fastidiosa strain IVIA5235, isolated from petiole tissues on PD2 medium after incubation at 28°C for 14 days of a sample from a symptomatic cherry tree identified in the first outbreak in Mallorca (2). Genomic DNA was recovered, using the DNeasy kit (Qiagen) for DNA extraction, from a pure culture grown on PD2 agar medium, and a whole-genome sequencing (WGS) library was prepared and paired-end sequenced by using an Illumina HiSeq 4000 platform.

Illumina sequencing yielded a total of 5,008,800 150-bp paired-end reads. The bacterial genome was reconstructed using the default settings of SPAdes v3.9.0 assembler (4) and consisted of 106 contigs, with an overall GC content of 51.6% and an *N*_50_ value of 103,359. A quality check of the assembly was performed by QUAST (5), and contigs not related to X. fastidiosa were discarded. In addition, a circular contig annotated as a plasmid sequence was obtained using plasmidSPAdes (6). The plasmid, named pXFAS_5235, consisted of 38,297 nucleotides and had a GC content of 49.2%, and a BLASTN search showed it has the highest sequence similarity with the conjugative plasmid pXFAS01 (GenBank accession number CP001012), which was reported in X. fastidiosa strain M23 (7). The average nucleotide coverages were 897× for pXFAS_5235 and 450× for the chromosomal genome of strain IVIA5235.

Functional annotation by submission to the NCBI Prokaryotic Genome Automatic Annotation Pipeline (PGAAP) (8) resulted in the identification of 48 tRNA loci, 2,430 genes, 2,258 protein-encoding genes in the chromosome, and 42 protein-encoding genes in the plasmid.

The occurrence of this plasmid in several hosts, infected by X. fastidiosa subsp. *fastidiosa* ST1, was successfully confirmed by conventional PCR using 2 distinct sets of primers amplifying a region of approximately 500 bp.

The draft genome of strain IVIA5235 further supports the genetic lineage of this strain as being close to X. fastidiosa subsp. *fastidiosa*. To our knowledge, this is the first sequenced genome of a European strain of X. fastidiosa subsp. *fastidiosa*, and the availability of this information will be critical for population genomics studies and molecular epidemiological investigations aiming at disclosing important aspects of the complex scenario in the Balearic Islands.

### Data availability.

The raw reads from this study have been submitted to the NCBI Sequence Read Archive (SRA) under accession number SRR7867948. The genome sequences of X. fastidiosa strain IVIA5235 have been submitted to the NCBI BioProject under accession number PRJNA488161 and deposited at DDBJ/ENA/GenBank under the accession number QWLC00000000. The version described in this paper is version QWLC01000000.
